# Asylum Treatment: What Is the Explanation?

**Published:** 1917-09-15

**Authors:** 


### ASYLUM TREATMENT: WHAT IS THE EXPLANATION?

A RECENT issue of the *Daily Graphic* contains a letter from a lady who signs herself "Under Certificate." She says that she was taken to an asylum as a voluntary boarder, but while there she was certified, that she subsequently again became a voluntary boarder, and finally left the asylum, presumably well. The terms and tones of the letter raise considerable doubt as to whether the lady really is recovered—it is such a contradictory and incoherent rhodomontade. On her own showing she entered the asylum of her own accord as a voluntary boarder, and yet she says that upon her arrival "sinister occurrences, unimaginable in their horror and humiliation, followed one another thick and fast, and my first night in the infirmary was one of inconceivable suffering and of maddening terror."

It will be noticed that all this is just in the vague, unparticularised phraseology of an unsound mind There is no hint of what the sinister occurrences were, or why they should have produced either horror or humiliation. There is nothing in the infirmary of an asylum, any more than in the wards of a hospital, to produce inconceivable suffering or maddening terror; and a person who confesses to such feelings confesses to disorder of mind.

The lady says that she was reported to be hypochondriacal, and gives us to understand that she was certified in consequence. She does not say to whom she was reported, nor how she knows that any report was made, nor does she give any other particular. As a fact, of course, hypochondria is not insanity, and the patient would certainly not be certified as insane because she was hypochondriacal. It seems, too, that her certification was the signal for her release, for she says: "Otherwise, I suppose, I must have been detained in that hell of hells until I had become delirious and raving, or even violent." It is as strange that certification should be the preliminary of discharge as it is that a patient should be certified as insane on account of hypochondria.

After she was certified it appears she had a visit from the chaplain, and then she was able to shake off the suspicion that there was any wish to or intention of certifying her. This, according to her own account, was after she had already been certified, and when she knew that by a mighty effort, or perhaps by a miracle, she had retained her sanity. She then goes on to speak of the "experts" lending themselves to so devilish a work as that of inducing certifiable symptoms in a sane woman placed in their hands for healing, and that these experts "callously perpetrated this horrible wrong as part of their day's work."

She says that her doctor informed her that one of the symptoms of insanity recorded against her was that she had asked for effervescent draughts; and she goes on to say that her case is not a usual one. Many are infinitely more disastrous and cruel. Perhaps they may be, but it is certain that, however, disastrous and cruel they may be, they could not be described in stronger terms. According to her own account, her treatment was a "hideous and dishonest farce"—"It was all a grinning lie!"

After this we are astounded to hear that a few cases profit by the existence of asylums, and that by these cases the asylum advertises itself. It is news to us that asylums advertise the cures that they effect. The whole tone of the letter suggests, in the first place, a certainty that the lady has been discharged too soon; and, in the second, an unbounded astonishment that a paper like the *Daily Graphic* should consent to publish such ravings.

